# High Genotypic Polymorphism and Extensive Gene Flow Drive the Population Dynamics of Potato Oomycete Pathogen, *Phytophthora infestans*, Across Southwestern China

**DOI:** 10.3390/jof12080553

**Published:** 2026-07-24

**Authors:** Xiaofeng Liu, Jun Chun, Hong Zhang, Zhonghan Fan, Yuhang Zhu, Qinghua Chen, Xiangquan Fan, Shaojiang Yang, Honghao Li, Xiaoli Wang

**Affiliations:** 1 Institute of Plant Protection, Sichuan Academy of Agricultural Sciences, Chengdu 610066, China; liuxf@scsaas.cn (X.L.); zhh503@163.com (H.Z.); fanzhonghan@scsaas.cn (Z.F.); zhuyuhang@scsaas.cn (Y.Z.); chenqinghua@scsaas.cn (Q.C.); 2 Chengdu Academy of Agriculture and Forestry Sciences, Chengdu 610072, China; jamiechun123@163.com; 3 Dazhou Academy of Agricultural Sciences, Dazhou 635000, China; dzfxquan@163.com; 4 Academy of Agricultural Sciences, Xichang 615000, China; yangsj2712@163.com

**Keywords:** potato late blight, *Phytophthora infestans*, population structure, multilocus genotyping, gene flow, fungicide (metalaxyl) resistance, mitochondrial haplotype, SSR markers

## Abstract

Potato late blight, *Phytophthora infestans*, remains a major global threat to potato production due to the rapid genetic change, long-distance dispersal and fungicide resistance. To clarify the genetic structure and phenotypic characteristics of *P. infestans* populations in southwestern China during 2019–2025, this study analyzed 241 isolates collected from Sichuan, Chongqing, Guizhou, Yunnan and Hubei. The isolates were characterized using 14 SSR markers, together with mating-type determination, metalaxyl sensitivity assays, mitochondrial haplotype identification and virulence profiling. A total of 70 alleles and 118 multilocus genotypes were detected, with certain genotypes (SW-40, SW-48 and SW-81) broadly distributed across regions, while the simultaneous presence of numerous low-frequency genotypes reflected high overall genetic diversity. Population genetic analyses revealed weak regional differentiation and strong genetic connectivity, with most genetic variation occurring within regional populations, where self-fertile isolates, metalaxyl-resistant phenotypes and mitochondrial haplotype Ia were predominant. Cluster and principal component analyses separated the isolates into three major genetic groups, with the largest group containing most isolates and showing close genetic similarity to the Blue_13 reference genotype. These findings indicate that southwestern China harbors a highly diverse and admixed *P. infestans* population, likely driven by frequent pathogen movement among potato-growing regions.

## 1. Introduction

Potato late blight, caused by the oomycete *Phytophthora infestans* (Mont.) de Bary (Peronosporaceae: Peronosporales), remains among the most destructive diseases affecting potato production worldwide, leading to substantial yield losses and economic burdens [1,2]. Under favorable environmental conditions, the pathogens can rapidly infect foliage and tubers, causing extensive crop damage and imposing high costs for fungicide application and crop protection measures [3]. Since the 1840s Irish potato famine, *P. infestans* has remained a main threat to global food security and continues to challenge potato production systems despite significant advances in breeding, disease forecasting and chemical and integrated control strategies [4]. The persistence of late blight as a major agricultural problem is largely attributed to the remarkable evolutionary and adaptive capacity of the pathogen, which enables populations to rapidly overcome host resistance, fungicide pressure, and environmental fluctuations. The epidemiology and management of potato late blight are strongly influenced by the genetic structure and diversity of *P. infestans* populations [5,6]. Over the past several decades, studies from Europe, North America, Asia and South America have documented substantial temporal, spatial and evolutionary variations in pathogen populations. In some regions, epidemics have been dominated by a limited number of highly successful clonal lineages, whereas in others, genetically diverse populations have been reported [5,7,8]. Such differences in population structure may critically affect pathogen dispersal, host adaptation, virulence evolution, and fungicide response [9]. Consequently, understanding the genetic diversity and connectivity among regional populations of *P. infestans* has become an essential component of disease surveillance and integrated management programs.

Several biological traits are commonly used to characterize *P. infestans* populations [10]. Mating-type composition provides essential insights into the reproductive potential of the pathogen, as the coexistence of A1 and A2 mating types enables sexual reproduction and the formation of long-lived oospores. In addition, self-fertile isolates have been increasingly reported in some populations and may further shape population dynamics [10,11]. Sensitivity to phenylamide fungicides, particularly metalaxyl, is another critical trait because resistant populations can significantly reduce the effectiveness of disease control programs and contribute to recurrent epidemics [12,13]. Mitochondrial DNA (mtDNA) haplotypes have also been widely used to investigate population history and lineage relationships, whereas virulence profiles on potato differential hosts provide information on the capacity of pathogen populations to overcome host resistance genes [14,15,16]. Molecular markers have substantially improved our ability to investigate the population biology of *P. infestans*. Among them, simple sequence repeat (SSR) markers are widely employed due to their high polymorphism, reproducibility and discriminative power. SSR-based analyses have been successfully applied to identify multilocus genotypes, evaluate genetic diversity, estimate gene flow and infer population structure in *P. infestans* populations from different regions of the world [12,17,18]. Integrating SSR genotyping with phenotypic and mitochondrial characteristics provides a comprehensive understanding of the factors shaping pathogen populations and their epidemiological consequences [19].

China is one of the world’s highest potato-producing countries and late blight remains a significant constraint to sustainable potato production [20]. Southwestern China, including Sichuan, Chongqing, Guizhou, Yunnan and Hubei, represents an important and diverse potato-growing region characterized by complex topography, variable climatic conditions, and extensive movement of seed and commercial potatoes among production areas [21,22]. These factors may facilitate pathogen dispersal and promote the development of genetically complex populations. Although several studies have examined the occurrence and characteristics of *P. infestans* in China, comprehensive information on the population biology in southwestern China remains limited [21,23,24]. Furthermore, the extent to which regional populations are genetically differentiated or connected through pathogen movement has not been fully resolved.

Understanding these population characteristics is critical for predicting pathogen spread, assessing the risk of fungicide resistance, and improving regional disease management strategies [9,10,13]. Therefore, this study investigated 241 *P. infestans* isolates collected from major potato-growing regions of southwestern China. Using SSR markers together with analyses of mating type, metalaxyl sensitivity, mitochondrial haplotype and virulence profile, we aimed to (I) characterize the genetic diversity and multilocus genotype composition of *P. infestans* populations, (II) assess population differentiation and connectivity among geographic regions, (III) determine the distribution of key phenotypic and mitochondrial traits and (IV) evaluate the relationships between genetic structure and biological characteristics. We hypothesized that frequent movement of potato planting materials among regions has contributed to the development of genetically connected populations despite geographic separations. The findings provide insights into the population biology of *P. infestans* and contribute to the development of more effective late blight monitoring and management programs in southwestern China.

## 2. Materials and Methods

### 2.1. P. infestans Growth Conditions

*Phytophthora infestans* isolates were maintained on rye B agar and incubated at 18 °C in darkness. Rye B agar was prepared using 60 g rye grains, 20 g sucrose and 15 g agar powder, with distilled water added to a final volume of 1000 mL. The prepared medium was autoclaved at 121 °C for 30 min before use.

### 2.2. Sampling and Isolation of P. infestans

A total of 241 *P. infestans* isolates were collected from major potato-growing areas in southwestern China, including Sichuan, Chongqing, Guizhou, Yunnan and Hubei. Potato leaves showing typical late blight lesions were sampled from 33 production areas. To avoid repeated sampling from the same lesion source, only one lesion was collected per leaflet, with sampling fields separated by at least 50 km and altitudes ranging from 324 to 3430 m. Detailed geographic information for each sampling location, including the number of isolates, latitude, longitude and altitude, is provided in Table 1. Sampling was performed during the main potato growing season (November/2019–2025) and all isolates were coded sequentially according to collection site and lesion number. Each leaf was wrapped in absorbent paper, placed in a kraft envelope and transported in a cool container. Within 48 h of collection, the leaves were washed five times with sterile ddH_2_O, air-dried briefly and placed upside down in humid infection trays for 20–30 h to promote mycelial growth. Each tissue piece was placed beneath a sterile slice of the susceptible potato cultivar Favorita, approximately 3 mm thick and incubated at 18 °C in darkness for 4–5 days. When mycelial growth appeared on the potato slices, hyphal tips were transferred to fresh rye B-agar medium to obtain pure cultures. Purification was achieved by re-growing mycelium on a new selective plate under the same conditions. Five mycelial plugs from each isolate were stored in milk in 2 mL tubes and subsequently preserved in liquid nitrogen (N_2_). Negative controls without inoculum were included to confirm aseptic conditions and cultures were monitored for purity for 5–7 days before further analysis.

### 2.3. Phenotypic Characterization

#### 2.3.1. Mating-Type Determination

The mating-type of each isolate was checked by dual co-cultivation with standard A1 (VK98014) and A2 (90128) tester isolates on rye-agar medium. Plates were incubated at 18 °C in darkness for 5–7 days. Interaction zones between colonies were examined microscopically for oogonia, antheridia and oospore formation and each isolate was scored for compatibility with each tester. Isolates that produced oospore when paired with the A1 tester were classified as A2 mating type, whereas isolates producing oospore when paired with the A2 tester were classified as A1. Isolates producing oospores with both tester isolates or in single culture were classified as self-fertile under the standardized in vitro assay [8]. Oospore formation was not evaluated in infected potato tissues; therefore, this classification does not directly confirm self-fertility in planta.

#### 2.3.2. Metalaxyl Sensitivity Assay

Metalaxyl sensitivity was determined using rye-agar medium amended with 5 μg/mL and 100 μg/mL metalaxyl, with unamended rye-agar as the control. Technical grade metalaxyl (Ridomil 25 WP, Novartis Agro AG, Basel, Switzerland) was dissolved in acetone and diluted with sterile water to prepare a stock solution, which was freshly prepared prior to each assay. Each *P. infestans* isolate was first grown on rye-agar for 7 days. A 5 mm diameter mycelial plug from the actively growing colony margin was transferred to the center of each test-plate. For each isolate and metalaxyl concentration, three replicate plates were prepared and colony diameter on each plate was measured along two perpendicular axes using the cross-method, with the mean of the two measurements used for subsequent analysis. Metalaxyl sensitivity was classified relative to the untreated control as resistant (relative growth ≥40% on both 5 μg/mL and 100 μg/mL plates), intermediate (≥40% only on 5 μg/mL plates), or sensitive (≤40% on both concentrations) [8,24,25].

#### 2.3.3. Physiological Race and Virulence-Gene Determination

Physiological races were determined using a detached-leaf assay on a standard set of potato differential hosts carrying major resistance genes R1–R11, together with a susceptible control lacking known R genes. Differential plants were maintained as sterile plantlets and grown under controlled conditions (temperature, light, humidity) to standardize leaf age and physiology. To prepare inoculum, approximately 1 cm^2^ of actively growing *P. infestans* culture was placed beneath a fresh sterile potato slice and incubated at 18 °C in darkness for 5 days. Mycelia were collected into 5 mL sterile water, gently shaken and filtered through one layer of gauze to obtain a sporangial suspension. The suspension was kept at 7 °C for 2–4 h to stimulate zoospore release and then adjusted to 5 × 10^4^ zoospores/mL. Differential plants were grown on MS medium for approximately 20 days and then transferred to coconut coir substrate for another 20 days and fully expanded leaflets were used for inoculation. Immediately after inoculation, the leaflets were maintained under high-humidity conditions in darkness for 12 h to facilitate infection and disease initiation. Thereafter, they were incubated at 18 °C under a 16 h photoperiod for disease development and disease reactions were assessed 7 days after inoculation. Infection was considered compatible when visible mycelia and sporangia were observed at the inoculation site, and resistant when no or only slight symptoms were present. Virulence genes (vir1–vir11) were assigned according to the ability of each isolate to infect the corresponding differential host. For each isolate–differential-host combination, the assay consisted of three replicates, with 10 leaflets included in each replicate, resulting in a total of 30 leaflets per combination.

### 2.4. P. infestans DNA Extraction

For DNA extraction, each isolate was cultured on fresh slices of the susceptible potato cultivar Favorita for 7 days and mycelia were harvested and stored at −20 °C until extraction. Genomic DNAs were isolated using the E.Z.N.A.^®^ Fungal DNA Mini Kit (Omega Bio-Tek, Inc., Norcross, GA, USA) following the manufacturer’s instructions. DNA quality as well as concentration were assessed using a NanoDrop ND-1000 spectro-photometer (NanoDrop Technologies, Wilmington, DE, USA). DNA samples were adjusted to 50 ng/μL and stored at −20 °C until SSR analysis.

### 2.5. SSR Genotyping

Fourteen SSR loci were used for genotyping: D13, G11, Pi04, Pi26, Pi4B, Pi4G, Pi63, Pi70, SSR2, SSR3, SSR4, SSR6, SSR8 and SSR11. Primer sequences and detailed information are provided in Table S1 [26,27,28]. PCR amplification was performed in a 25 μL reaction volume containing 2.0 μL genomic DNA, 0.5 μL forward primer, 0.5 μL reverse primer, 0.5 μL dNTPs, 2.5 μL 10× PCR buffer, 2.0 μL MgCl_2_, 0.2 μL Taq-DNA polymerase and 16.8 μL ddH_2_O. PCR amplification was conducted under the following conditions: initial denaturation at 95 °C for 15 min; 30 cycles of denaturation at 95 °C for 30 s, annealing at 58–60 °C for 90 s and extension at 72 °C for 60 s; followed by a final extension at 72 °C for 20 min. PCR product was checked by 1% agarose-gel electrophoresis and then analyzed using an ABI 3730XL DNA-analyzer (Applied Biosystems, Foster City, CA, USA). Allele sizes were scored using GeneMapper (Ver. 4.0.) and SSR allele data were converted into multilocus allele profiles and a binary matrix for subsequent genotype and population genetic analyses. The reference isolate NL05246, representing the Blue_13 genotype, was included in all analyses for comparative purposes.

### 2.6. Mitochondrial Haplotype Determination

Mitochondrial DNA (mtDNA) haplotypes were determined for the tested isolates and classified as Ia, IIa or IIb according to established *P. infestans* mtDNA haplotype categories. Haplotype data were used to compare mitochondrial lineage composition among regions, mating-type groups and genetic clusters. PCR amplification of mtDNA regions was performed in duplicate for each isolate to confirm reproducibility and negative controls without DNA template were included to monitor contamination. Alleles were scored using the ABI 3730XL DNA analyzer and GeneMapper software and haplotype assignments were independently verified by two researchers [29].

### 2.7. Population Genetic and Statistical Analyses

SSR data were used to identify multilocus genotypes among the 241 *P. infestans* isolates. Genotype frequency, regional genotype distribution, shared genotypes and unique genotypes were calculated while a genotype detected only once was defined as a rare genotype. Normalized Shannon’s diversity index was calculated to compare genotype diversity among regional populations and mating-type groups. Genetic diversity was assessed using allele number, effective allele number, observed heterozygosity, expected heterozygosity, Shannon information index, polymorphic information content and fixation indices. Hardy–Weinberg equilibrium was tested for each SSR locus and population differentiation was estimated using FST and PhiPT values. Gene flow among populations was estimated using the formula Nm = 0.25(1 − FST)/FST. Analysis of molecular variance (AMOVA) was performed to partition genetic variation within and among geographic populations and pairwise PhiPT values and gene flow estimates were calculated among all regional populations. Nei’s genetic distance and genetic identity were computed to evaluate genetic relationships and clustering analyses were conducted using Euclidean distance and the unweighted pair-group method with arithmetic mean (UPGMA). Principal component analysis (PCA) was performed to visualize genetic structure among isolates and to compare the genetic clustering pattern with the UPGMA-based grouping. Phenotypic and genetic traits, including mating type, metalaxyl sensitivity, mtDNA haplotype, virulence-gene profile, SSR genotype, and regional origin, were summarized as frequencies or percentages where appropriate. All analyses were performed using R software version 4.0.2 (R Core Team, 2017).

## 3. Results

### 3.1. SSR Marker Polymorphism Analysis

The 14 SSR loci generated a total of 70 alleles among the 241 *P. infestans* isolates collected from southwestern China (Table 2). The number of alleles per locus ranged from 1 to 11, with an average of five alleles per locus. D13 exhibited the highest polymorphism, with 11 alleles and a polymorphic information content (PIC) value of 0.81, followed by G11, whereas Pi70 was monomorphic. The mean observed heterozygosity (H_o_ = 0.65) was higher than the mean expected heterozygosity (H_e_ = 0.47), indicating an overall excess of heterozygotes. Except for Pi70 and SSR2, all loci significantly deviated from Hardy–Weinberg equilibrium (*p* < 0.05), indicating a non-random population structure.

Regional comparisons also revealed considerable genetic diversity (Table 3). Sichuan showed the highest allelic richness and Shannon’s information index, whereas average heterozygosity was broadly comparable among the five regional populations. The proportion of polymorphic loci ranged from 71.43% in Guizhou to 92.86% in Sichuan and Hubei. In all regional populations, observed heterozygosity exceeded expected heterozygosity, resulting in negative FIS values. Collectively, these results indicate substantial genetic diversity and heterozygote excess across the regional populations.

### 3.2. Multilocus Genotype Composition and Regional Distribution

**Figure 1:**
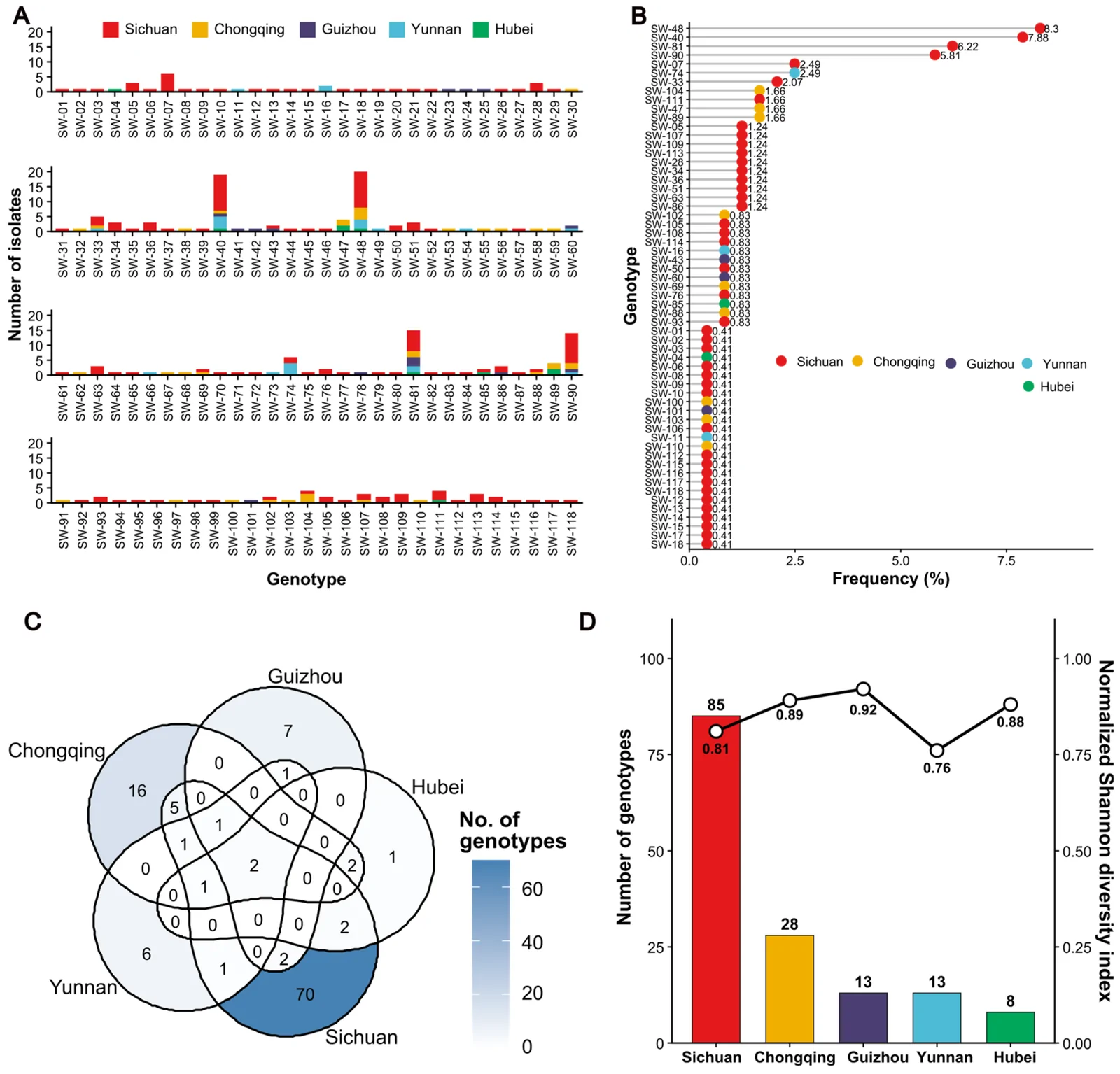
Multilocus SSR genotype composition and regional distribution of *P. infestans* populations. (**A**) Distribution of SSR genotypes among regional populations. (**B**) Frequency of dominant SSR genotypes across regions. (**C**) Number of SSR genotypes and normalized Shannon’s diversity index in each region. (**D**) Shared and unique SSR genotypes among the five regional populations.

Multilocus SSR analysis revealed extensive genotype diversity among the 241 *P. infestans* isolates. In total, 118 SSR genotypes were identified, indicating that the regional population was composed of numerous genetically distinct lineages rather than a single dominant clonal group (Figure 1). Genotype frequencies were unevenly distributed, with a few relatively common genotypes occurring across multiple regions and a large number of genotypes represented by only one isolate. Among all detected genotypes, SW-48 was the most frequent, accounting for 8.30% of the total population. This genotype was detected in Sichuan, Chongqing, Yunnan and Hubei, showing that it was widely distributed but not present in every sampled region. SW-40 was the second most frequent genotype, accounting for 7.88% of isolates and was detected in all five regions: Sichuan, Chongqing, Guizhou, Yunnan and Hubei. SW-81 was also particularly important because, together with SW-40, it occurred in all five regions. These two genotypes had a combined frequency of 14.11%, highlighting that several multilocus genotypes are broadly shared across southwestern China, suggesting frequent gene flow among regions.

Region-specific genotype patterns were also evident in Sichuan; 85 SSR genotypes were detected, with SW-40 and SW-48 being the most frequent genotypes, each representing 7.69% of the Sichuan population. In Chongqing, 28 genotypes were detected and SW-48 was the most frequent genotype, accounting for 10.81% of isolates from this region. In Guizhou, 13 genotypes were detected, with SW-81 showing the highest frequency at 20.00%. In Yunnan, 13 genotypes were detected and SW-40 and SW-74 were the most frequent, each accounting for 17.39% of the Yunnan population. In Hubei, 8 genotypes were identified, with SW-47 and SW-89 being the most frequent, each representing 20.00% of isolates from this region. These regional differences demonstrate that, although some genotypes are widely shared, each population also maintains distinct dominant genotypes, reflecting localized diversification (Figure 1A,B).

Rare genotypes contributed strongly to the overall diversity of the *P. infestans* populations. Among the 118 detected SSR genotypes, 84 were represented by only one isolate, accounting for 71.19% of all genotypes. These rare genotypes were distributed across the five sampled regions, suggesting continuous diversification or repeated introduction of genetically distinct lineages over time. The number of unique genotypes also varied among regions, with Sichuan containing the largest number of genotypes (70), followed by Chongqing (16), Guizhou (7), Yunnan (6) and Hubei (1). These patterns indicate that Sichuan serves as the major reservoir of *P. infestans* genotype richness in southwestern China (Figure 1C and Figure S1A). Normalized Shannon diversity indices further supported the presence of high genotype diversities within regional populations. The overall normalized Shannon diversity index for southwestern China was 0.78 and among regions, genotype diversity was highest in Guizhou (Hs = 0.92), followed by Chongqing (Hs = 0.89), Hubei (Hs = 0.88), Sichuan (Hs = 0.81) and Yunnan (Hs = 0.76). Although Sichuan contained the largest number of isolates and genotypes, the high normalized diversity values in smaller populations such as Guizhou and Hubei indicate that genotype diversity was not solely dependent on sample size, but reflects true genetic variation within populations (Figure 1D).

The regional distribution of isolates was uneven, with Sichuan contributing the largest proportion, followed by Chongqing, Yunnan, Guizhou and Hubei (Figure S1B). Genotype sharing was also observed among mating-type groups (Figure S2). A1, A2 and self-fertile isolates showed different levels of genotype richness, but several multilocus genotypes were shared between A1 and self-fertile isolates. This indicates that identical SSR backgrounds can occur across different reproductive phenotypes, highlighting the potential for recombination or clonal spread among mating types. Overall, the coexistence of widespread genotypes demonstrates that *P. infestans* populations in southwestern China are both highly diverse and strongly connected across geographic regions.

### 3.3. Phenotypic Variation and Mitochondrial Haplotype Structure

**Figure 2:**
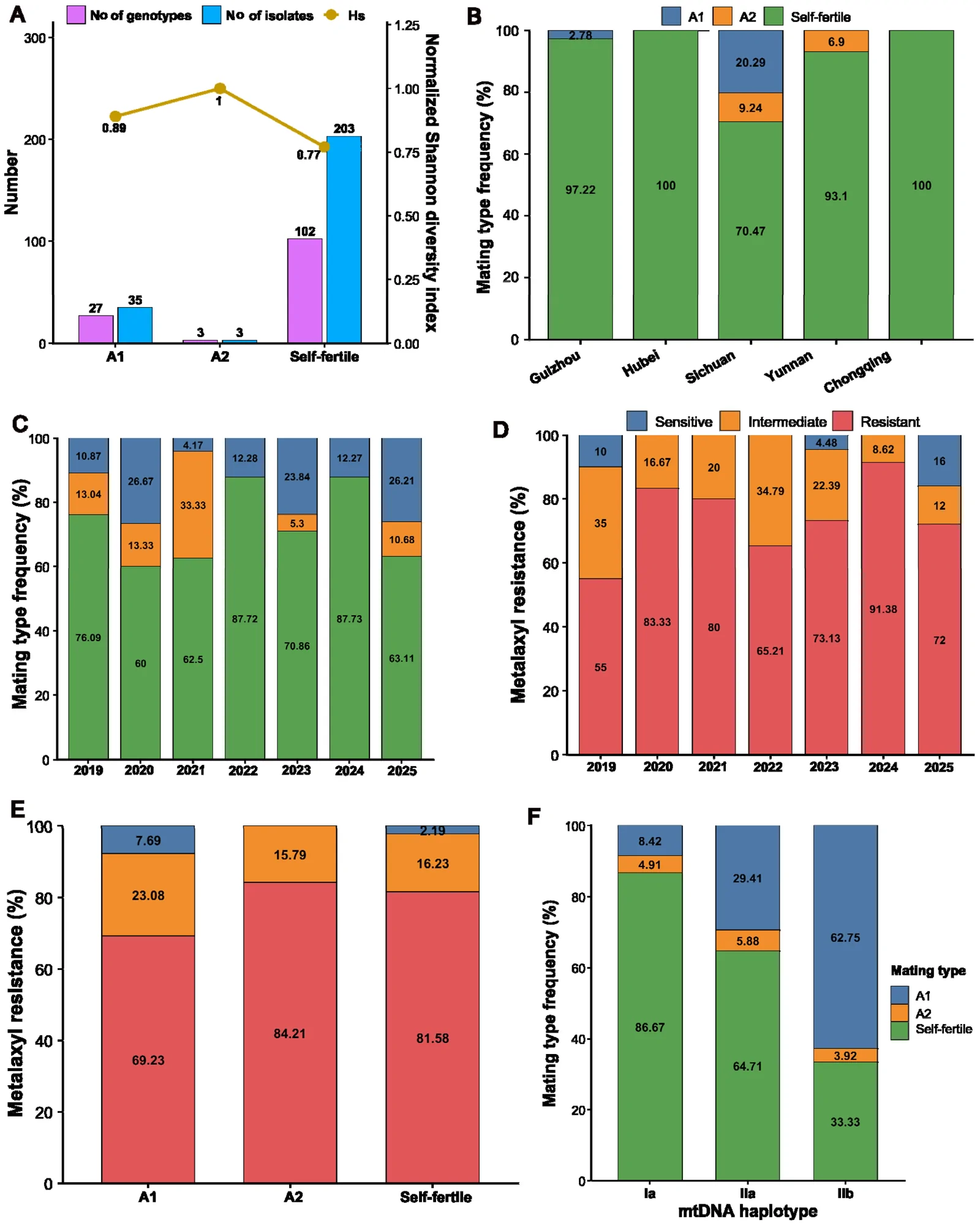
Phenotypic and mitochondrial haplotype variation in *P. infestans* populations. (**A**) No. of isolates, no. of SSR genotypes and normalized Shannon’s diversity index among mating-type groups. (**B**) Regional distribution of mating types. (**C**) Temporal distribution of mating types across sampling years. (**D**) Temporal distribution of metalaxyl sensitivity phenotypes. (**E**) Metalaxyl sensitivity distribution among mating-type groups. (**F**) Association between mtDNA haplotypes and mating types. A1 and A2 indicate mating types. S, sensitive; I, intermediate; R, resistant. Ia, IIa and IIb indicate mtDNA haplotypes.

Phenotypic characterization showed that the *P. infestans* population from southwestern China was dominated by self-fertile isolates, but A1 and A2 mating types were also detected (Figure 2A). Among the 241 isolates, self-fertile isolates represented the largest reproductive group, followed by A1 isolates, whereas A2 isolates occurred at very low frequencies. The self-fertile group also contained the largest number of isolates and multilocus genotypes. However, normalized Shannon diversity was higher in the A1 and A2 groups than in the self-fertile group, indicating that even smaller mating-type groups retain considerable genotypic diversity and may contribute disproportionately to the overall population heterogeneity. Also, the regional distribution of mating types showed clear differences among populations as Self-fertile isolates were detected across the sampled regions and represented the dominant mating-type category in most regional populations. A1 isolates were also present in multiple regions but occurred at lower frequencies than self-fertile isolates. A2 isolates were rare and were detected only in a limited part of the dataset. These patterns indicate that the population is composed of multiple reproductive phenotypes, with self-fertility being predominant but not exclusive, suggesting the coexistence of sexual and asexual reproduction in southwestern China (Figure 2B).

Temporal variations in mating-type composition were also observed across different sampling years. Self-fertile isolates remained the dominant category in most years, whereas A1 and A2 isolates occurred less consistently. The persistence of self-fertile isolates over multiple years demonstrates that this reproductive phenotype is a stable component of the regional population rather than a transient occurrence (Figure 2C). Metalaxyl sensitivity assays revealed that resistant isolates were overwhelmingly dominant in the populations. Most isolates were classified as resistant, whereas intermediate isolates occurred at lower frequency and sensitive isolates were rare. Temporal data show that resistant isolates were consistently present across all sampling years, indicating that metalaxyl resistance is widespread and persistent in the regional populations (Figure 2D). Moreover, the relationship between mating type and metalaxyl sensitivity showed that resistance was present across reproductive groups. Self-fertile isolates contained the largest number of resistant isolates because they represented the dominant mating-type group in the collection. A1 isolates also included resistant individuals, whereas sensitive isolates were rare across all mating-type categories (Figure 2E). These results indicate that metalaxyl resistance is not restricted to a single mating type but is broadly distributed across the population, highlighting the adaptive potential of these isolates.

Mitochondrial haplotype analysis showed that Ia haplotype was the predominant mtDNA type, particularly among self-fertile isolates. The IIa and IIb occurred at lower frequencies and showed more restricted distributions (Figure 2F). The regional mtDNA haplotype pattern further showed that haplotype composition differed among populations, but Ia remained the major haplotype in most regions (Figure S3). Furthermore, the temporal distribution of virulence genes showed that multiple virulence factors were present during the sampling period, with some virulence genes occurring at high frequency across years and others showing more variable annual patterns (Figure S4). These patterns indicate that the pathogen population maintains diverse virulence combinations alongside high SSR genotype diversity, reinforcing the complexity and adaptive potential of *P. infestans* in southwestern China. Overall, the coexistence of widespread genotypes, multiple reproductive phenotypes, metalaxyl resistance and mitochondrial haplotype variation demonstrates that *P. infestans* populations in southwestern China are highly diverse and strongly connected across geographic and temporal scales. This comprehensive phenotypic and genotypic diversity underscores the need for integrated disease management strategies that consider both genetic structure and reproductive capacity.

### 3.4. Genetic Differentiation, Gene Flow and Population Connectivity

Population genetic analysis based on the 14 SSR loci showed low genetic differentiation among regional *P. infestans* populations. Across loci, FST values ranged from 0.0000 to 0.0812, with an overall FST of 0.0244, indicating that only a small proportion of genetic variation was attributable to differentiation among regions. Most loci showed low FST values, whereas SSR3 had the highest FST value (0.0812), suggesting relatively greater differentiation at this locus. In contrast, Pi04 and Pi70 showed no detectable differentiation among populations. Estimated gene flow (Nm) values varied among loci, but most were greater than 1, indicating substantial genetic exchange among regional populations (Table 4). The fixation indices also indicated heterozygote excess in the regional population as most loci showed negative FIS and FIT values, with total FIS and FIT values of −0.4436 and −0.4158, respectively. These negative values indicate that observed heterozygosity exceeded expected heterozygosity across much of the dataset, consistent with a genetically admixed population containing excess heterozygotes, while loci such as Pi4G and SSR3 show positive FIS or FIT, reflecting localized deviations.

The analysis of molecular variance further confirmed that genetic variation was mainly distributed within regions rather than among regions. Variation among regional groups accounted for only 2.19% of the total molecular variance, whereas variation within groups accounted for 97.81%. These distributions indicate that isolates from different regions were genetically similar at the population level and that most genetic diversity was retained within local populations (Table 5). Furthermore, the pairwise PhiPT values among regional populations were generally low, ranging from 0.000 to 0.043. The highest pairwise differentiation was observed between Chongqing and Yunnan, whereas very low differentiation was observed between Sichuan and Hubei. These PhiPT estimates further support strong connectivity among regions, as the highest estimated gene flow occurred between Sichuan and Hubei, followed by Sichuan and Chongqing, whereas Chongqing and Yunnan showed the lowest estimated gene flow among pairwise comparisons (Table 6). Nei’s genetic distance and genetic identity showed the close relationship among regional populations (Table 7). Genetic distances ranged from 0.019 to 0.052, while genetic identity values ranged from 0.949 to 0.982. The shortest genetic distance was observed between Sichuan and Chongqing, indicating the closest genetic relationship between these two populations. The greatest genetic distance was observed between Guizhou and Yunnan, although the value remained low overall reflecting minimal divergence across all populations. Together, the low FST, low PhiPT, high within-population variance, high genetic identity and elevated gene flow indicate that *P. infestans* populations in southwestern China are highly connected and weakly differentiated at regional scales.

### 3.5. Integrated Cluster Structure of Genotypic and Phenotypic Profiles

**Figure 3:**
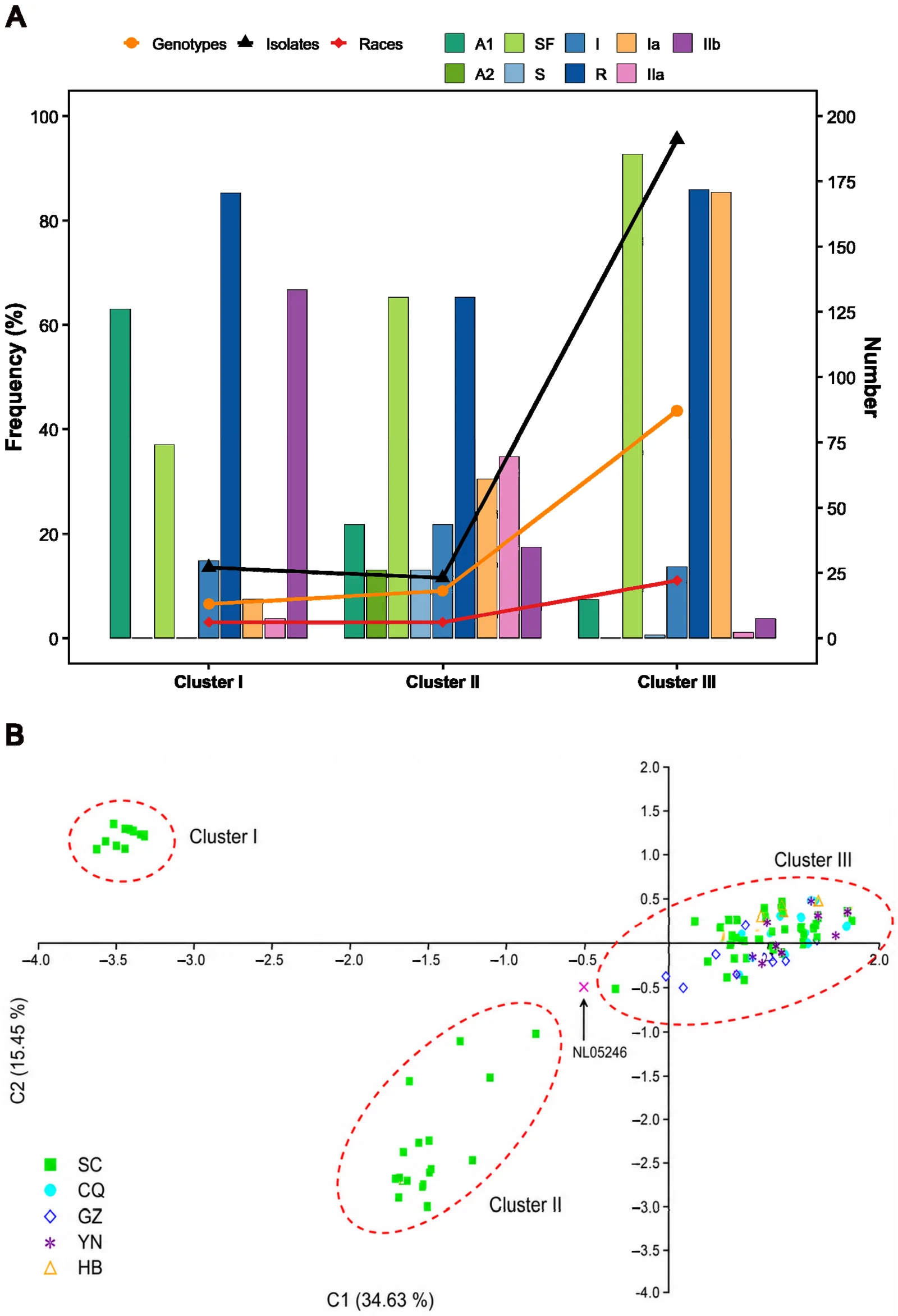
Integrated cluster structure of genotypic and phenotypic profiles in *P. infestans* populations. (**A**) Distribution of mating type, metalaxyl-sensitivity, mtDNA haplotype, physiological race number, isolate number and genotype number among the three genetic clusters. (**B**) PCA showing the genetic structure of isolates based on SSR data. Dashed ellipses indicate the three major genetic clusters. NL05246 represents the reference Blue_13 genotype. SC, Sichuan; CQ, Chongqing; GZ, Guizhou; YN, Yunnan; HB, Hubei.

Cluster-based analysis separated the 241 *P. infestans* isolates into three major genetic groups and these groups differed in genotype composition, mating type, metalaxyl sensitivity, mitochondrial haplotype and physiological race structures (Figure 3A). Cluster III was the predominant group, containing 191 isolates, whereas Cluster I and Cluster II contained 27 and 23 isolates, respectively, reflecting both regional and phenotypic structuring within the population. Cluster I contained 27 isolates and 13 SSR genotypes, dominated by A1 mating type, metalaxyl-resistant isolates and mtDNA haplotype IIb. No sensitive isolates were detected in this cluster, highlighting its unique phenotypic and mitochondrial profile.

Cluster II contained 23 isolates and 18 SSR genotypes, with a high proportion of self-fertile isolates and mixed Ia, IIa and IIb haplotypes. Resistant isolates were still dominant, with a small number of intermediate and sensitive individuals. Cluster III represented the core genetic background of the regional population, containing 191 isolates, 87 SSR genotypes and 22 physiological races, dominated by self-fertile phenotype, metalaxyl resistance and haplotype Ia. This cluster appears to capture the main regional genetic diversity, serving as the central reservoir of genotypic and phenotypic variation (Figure 3A).

PCA supported the separation of the three major genetic groups identified by cluster analysis (Figure 3B). Isolates assigned to Cluster I, Cluster II and Cluster III formed distinguishable groups in the PCA space, although Cluster II was positioned closer to Cluster III than to Cluster I. This pattern suggests that Cluster III represents the main regional genetic background, while Cluster I is more genetically distinct and may reflect localized lineages or recent introductions. The reference isolate NL05246, representing the Blue_13 genotype, was positioned close to Cluster III, but no tested isolate showed a completely identical SSR profile to the reference genotype. UPGMA analysis further corroborated this three-cluster structure, with the majority of isolates grouping within Cluster III (Figure S5). Overall, these results demonstrate that the genetic structure of southwestern Chinese *P. infestans* populations is associated with key phenotypic traits, including mating type, metalaxyl-sensitivity, mitochondrial haplotype and physiological race diversities. The integrated analysis of genotypic and phenotypic profiles highlights the coexistence of dominant and rare lineages, suggesting both clonal expansion and local diversification within regional populations.

## 4. Discussion

The population structure of *P. infestans* plays a central role in the epidemiology and management of potato late blight because genetic diversity influences pathogen adaptation, dispersal, fungicide sensitivity and the durability of host resistance [9,10]. Understanding the composition, genetic diversity, and gene flow among regional populations is therefore essential for disease surveillance and the development of effective management strategies [8]. By integrating SSR genotyping with analyses of mating type, metalaxyl sensitivity, mitochondrial haplotype and virulence characteristics, the present study provides a comprehensive assessment of *P. infestans* populations in the major potato-growing regions of southwestern China, 2019–2025.

Our study revealed a high level of genetic diversity detected within the regional population of *P. infestans*. SSR analysis identified 118 multilocus genotypes among 241 isolates, with a large proportion represented by unique or low-frequency genotypes. This high genotype diversity suggests the coexistence of multiple evolutionary lineages and substantial standing genetic variation, providing the population with significant adaptive potential. Similar patterns of diversity have been reported in other *P. infestans* populations worldwide and are often associated with long-term persistence, repeated introductions, or the coexistence of multiple evolutionary lineages [7,30]. In contrast to populations dominated by a single aggressive clonal lineage, the southwestern Chinese population appears to contain a broad spectrum of genetic backgrounds. The high values of heterozygosity and genotype diversity observed further support the view that the regional population is genetically complex and capable of maintaining substantial evolutionary potential [31]. Despite the presence of numerous low-frequency genotypes, several multilocus genotypes were widely distributed among geographic regions. The occurrence of genotypes such as SW-40, SW-48 and SW-81 across multiple provinces indicates that certain lineages possess a strong capacity for regional dissemination, likely facilitated by the movement of infected potato tubers and active regional trade. At the same time, many genotypes were restricted to individual regions, particularly in Sichuan, which contained the largest number of isolates and genotypes. The coexistence of widespread and region-specific genotypes reflects the combined effects of local diversification and regional dispersal, shaping population structure across southwestern China [32,33].

Population genetic analyses consistently indicated weak differentiation among regional populations. Low FST and PhiPT values, together with high gene flow estimates and the analysis of variance results indicated that genetic variation was mainly distributed within regional populations, implying weak genetic isolation among regions [34]. These findings are consistent with the hypothesis that frequent exchange of infected planting materials facilitates the movement of pathogen genotypes across potato-growing regions [35,36,37]. The widespread occurrence of several dominant genotypes across multiple provinces further supports these interpretations [33]. Compared with previous reports from other parts of China, the southwestern population examined here showed weaker regional differentiation and stronger genetic connectivity, whereas earlier studies identified clearer geographic clustering and distinct regional lineages [21,23,33]. A high frequency of self-fertility has also been reported in Gansu [20], indicating that this trait is not unique to southwestern China. These regional differences may reflect variation in seed-potato movement, cropping systems, climatic conditions, host cultivars and fungicide selection pressure. The mating-type composition of a pathogen population can provide important insights into its reproductive biology and evolutionary potential [38,39,40]. In our study, self-fertile isolates represented the dominant reproductive group, whereas A1 isolates were less common and A2 isolates occurred at relatively low frequencies. The predominance of self-fertile isolates is epidemiologically important because they can produce oospores without a compatible mating partner, increasing survival under unfavorable conditions [41,42] and enabling long-term persistence in agricultural systems [43]. The predominance of self-fertile isolates may contribute to population persistence by allowing oospore formation without the presence of the opposite mating type under suitable conditions. The subsequent clonal multiplication and movement of these isolates through infected planting materials may help maintain widely distributed genotypes and reduce genetic differentiation among regions. However, the independent contribution of self-fertility cannot be separated from clonal propagation and pathogen migration using the present SSR data. Although the low frequency of A2 isolates may limit opportunities for widespread sexual recombination, the coexistence of multiple mating types and high genotypic diversity indicates that evolutionary potential is not restricted, and adaptation to selective pressures remains possible [44]. Furthermore, the sharing of multilocus genotypes among mating-type groups suggests that reproductive phenotype and SSR genotype are not strictly associated and that similar genetic backgrounds may occur across different reproductive categories.

The widespread occurrence of metalaxyl-resistant isolates represents another important characteristic of the *P. infestans* population in southwestern China. Phenylamide fungicides have played an important role in late blight management for several decades [45,46]; however, the emergence and spread of resistant populations have reduced their effectiveness in many potato growing regions [13,47,48]. Resistant isolates predominated across sampling years, regions and mating-type groups, indicating that metalaxyl resistance is broadly established within the population rather than restricted to a few lineages. This pattern suggests that fungicide resistance may have become established within the population and could persist even in the absence of strong selective pressure [49]. Mitochondrial haplotype analysis revealed the predominance of haplotype Ia within the regional population, whereas haplotypes IIa and IIb occurred at lower frequencies. Mitochondrial haplotypes have frequently been used to investigate lineage relationships and historical population changes in *P. infestans* [4]. The distribution of mitochondrial haplotypes among clusters and mating types indicates that population structure cannot be inferred from a single genetic marker alone. Instead, the population appears to be shaped by a combination of historical introductions, pathogen dispersal, and subsequent diversification [50,51].

Virulence diversities represent another important component of pathogen adaptation because it influences the ability of *P. infestans* to overcome host resistance genes [22,52]. Multiple virulence factors were present across sampling years, indicating coexistence of diverse pathogenic phenotypes. Such diversity increases the probability that some genotypes can infect cultivars carrying specific resistance genes, complicating disease management [15,16]. Cluster-based analyses provided additional insights into population structure of *P. infestans* [53]. Three major genetic groups were identified, with the largest cluster containing most isolates, genotypes and physiological races. These patterns demonstrate that a substantial portion of the population shares a common genetic background, while significant phenotypic and genotypic variation persists. The PCA and UPGMA analyses showed generally consistent clustering patterns, supporting the robustness of the inferred population structure [53]. Interestingly, the Blue_13 reference isolate was positioned near the largest cluster, indicating partial genetic similarity to this globally important lineage; however, none of the southwestern Chinese isolates exhibited an identical SSR profile, supporting their regional genetic distinctiveness [22,37,44].

Taken together, these findings suggest that the population structure of *P. infestans* in southwestern China is shaped by the interaction of multiple evolutionary and epidemiological processes [22]. High genotype diversity indicates the presence of substantial standing genetic variation, whereas low differentiation and high gene flow indicate extensive regional connectivity [32,54,55]. From a practical perspective, the extensive connectivity among regional populations suggests that disease management strategies should be coordinated at regional scales, incorporating surveillance, seed-tuber certification and continuous monitoring of fungicide resistance and pathogen diversity to limit the spread of aggressive or resistant lineages.

## 5. Conclusions

This study characterized the genetic and phenotypic structure of *P. infestans* populations in southwestern China. SSR analysis revealed high genotypic diversity, weak regional differentiation and substantial genetic similarity among populations from different potato-growing regions. Self-fertile isolates, metalaxyl-resistant phenotypes and mitochondrial haplotype Ia were predominant, while cluster analyses identified three major genetic groups. These patterns are consistent with extensive regional connectivity and may reflect the combined effects of pathogen dispersal, local persistence and movement of infected potato planting material. However, because pathogen movement was not directly measured, this interpretation should be considered an epidemiological inference based on the observed genetic patterns. The findings provide a basis for regional surveillance, resistance monitoring, and coordinated late blight management.

## Figures and Tables

**Table 1:** Geographic origin and sampling information of *P. infestans* collections used in this study.

Region	Location	No. of Isolates	Latitude	Longitude	Altitude
Guizhou	Weining	9	26°83′	104°24′	2169
	Chishui	6	28°47′	105°76′	299
Hubei	Lichuan	10	30°23′	108°99′	1129
Chongqing	Kaizhou	15	31°20′	108°34′	529
	Shizhu	4	29°99′	108°11′	553
	Wuxi	13	31°41′	109°56′	256
	Yunyang	3	31°36′	108°91′	1280
	Zhongxian	2	30°29′	108°03′	189
Sichuan	Beichuan	6	31°94′	104°41′	996
	Chaotian	11	32°62′	106°10′	1412
	Chongzhou	7	30°54′	103°65′	508
	Danling	6	29°98′	103°36′	485
	Daofu	7	30°48′	101°48′	3430
	Ebian	11	29°24′	103°18′	1197
	Jiangyou	9	31°97′	104°78′	588
	Leibo	7	28°39′	103°77′	1118
	Luding	4	29°64′	102°12′	1562
	Mianning	9	28°74′	102°25′	2076
	Pengzhou	5	31°21′	103°78′	1050
	Puge	1	27°48′	102°48′	1417
	Shunqing	3	31°05′	106°13′	324
	Tongjiang	5	32°47′	107°37′	1200
	Wanyuan	7	32°11′	108°10′	1044
	Xiaojin	13	31°08′	102°30′	3162
	Xide	9	28°30′	102°45′	2410
	Xindu	8	30°77′	104°21′	478
	Xingwen	8	28°27′	105°29′	399
	Xuanhan	7	31°35′	107°72′	297
	Yilong	5	30°87′	106°08′	313
	Zhaojue	8	28°01′	102°51′	2600
Yunnan	Daguan	5	27°74′	103°89′	1135
	Huize	8	26°43′	103°32′	2129
	Ludian	10	27°17′	103°58′	1908
Total		241			

Note: Latitude and longitude coordinates indicate the approximate geographic location of each sampling site.

**Table 2:** Profile of SSR loci and variability in *P. infestans* populations.

Locus	N	N_a_	N_e_	H_O_	H_E_	I	PIC	*p* Val. (HWE)
D13	241	11	5.54	0.90	0.82	1.88	0.81	0.00
G11	241	7	3.27	0.91	0.70	1.43	0.69	0.00
Pi04	241	2	2.00	1.00	0.50	0.69	0.50	0.00
Pi26	241	7	2.77	0.93	0.64	1.23	0.64	0.00
Pi4B	241	5	2.22	0.88	0.55	0.90	0.55	0.00
Pi4G	241	8	3.12	0.54	0.68	1.45	0.68	0.00
Pi63	241	2	1.97	0.87	0.49	0.69	0.50	0.00
Pi70	241	1	1.00	0.00	0.00	0.00	0.00	NA †
SSR2	241	3	1.11	0.09	0.10	0.21	0.16	0.87
SSR3	241	5	1.37	0.12	0.27	0.56	0.33	0.00
SSR4	241	7	2.42	0.94	0.59	1.09	0.59	0.00
SSR6	241	5	2.02	0.83	0.51	0.80	0.52	0.00
SSR8	241	3	2.19	0.90	0.54	0.86	0.55	0.00
SSR11	241	4	1.26	0.20	0.21	0.44	0.31	0.00
Mean	241	5	2.30	0.65	0.47	0.87	0.49	

Note: N, number of *P. infestans* isolates; N_a_, number of alleles; N_e_, effective number of alleles; H_o_, observed heterozygosity; H_E_, expected heterozygosity; I, Shannon’s information index; HWE, Hardy–Weinberg equilibrium; NA †, Monomorphic locus, test not applicable.

**Table 3:** Genetic diversity of *P. infestans* populations from different regions.

Populations	N	PL	*P* (%)	N_a_	N_e_	I	H_o_	H_E_	$\bar{H}$	F_IS_
Sichuan	156	13	92.86	4.86	2.41	0.93	0.64	0.49	0.57	−0.306
Chongqing	37	12	85.71	2.33	2.12	0.73	0.69	0.43	0.56	−0.605
Guizhou	15	10	71.43	2.36	1.90	0.61	0.66	0.40	0.53	−0.650
Yunnan	23	11	78.57	2.29	1.98	0.61	0.70	0.40	0.55	−0.750
Hubei	10	13	92.86	3.07	2.19	0.79	0.66	0.49	0.58	−0.347
Overall population	241	13	92.86	5.00	2.31	0.87	0.65	0.47	0.56	−0.383

Note: N, number of isolates; PL, number of polymorphic SSR loci; *P* (%), percentage of polymorphic loci; N_a_, mean number of alleles per locus; N_e_, mean effective number of alleles per locus; I, mean Shannon’s information index; H_o_, mean observed heterozygosity; H_E_, mean expected heterozygosity; $\bar{H}$, average heterozygosity; and F_IS_, fixation index. PL was calculated as the number of polymorphic loci among the 14 SSR loci within each regional population, with monomorphic loci excluded. Na, Ne, I, Ho, H_E_, $\bar{H}$ and F_IS_ were calculated across all loci for each population. In the “Overall population” row, N represents the total number of isolates, whereas the remaining genetic-diversity statistics represent values calculated for the pooled population across all loci.

**Table 4:** Population genetic parameters for 14 SSR loci in *P. infestans* populations.

Locus	Fis	Fit	F_ST_	Nm
D13	−0.1970	−0.1727	0.0204	12.0335
G11	−0.4371	−0.3717	0.0455	5.2429
Pi04	−1.0000	−1.0000	0.0000	NA †
Pi26	−0.6646	−0.6462	0.0110	22.4786
Pi4B	−0.7324	−0.7240	0.0048	51.4041
Pi4G	0.0508	0.1086	0.0609	3.8522
Pi63	−0.8785	−0.8714	0.0038	65.8159
Pi70	NA †	NA †	0.0000	NA †
SSR2	−0.0083	0.0183	0.0265	9.2001
SSR3	0.5249	0.5635	0.0812	2.8277
SSR4	−0.7657	−0.7472	0.0105	23.5071
SSR6	−0.7947	−0.7692	0.0142	17.3765
SSR8	−0.8216	−0.8067	0.0082	30.2037
SSR11	−0.0432	0.0133	0.0542	4.3657
Total	−0.4436	−0.4158	0.0244	20.6923

Note: Fis, inbreeding coefficient within populations; Fit, overall inbreeding coefficient across the total population; F_ST_ represents genetic differentiation coefficient; Nm represents gene flow. NA †, monomorphic locus, parameters not applicable.

**Table 5:** Analysis of molecular variance among and within regional *P. infestans* populations.

Source	df	SS	MS	Est. Var.	(%)
Between groups	4	27.760	6.940	0.090	2.19
Within groups	236	945.896	4.008	4.008	97.81
Total	240	973.656		4.098	100

Note: df, degrees of freedom; SS, sum of squares; MS, mean squares; Est. Var., estimated variance component, (%) indicates the proportion of total molecular variance explained by variation among or within populations. Data were calculated based on 14 SSR loci across 241 isolates.

**Table 6:** Pairwise PhiPT values and gene flow among regional *P. infestans* populations.

Populations	Sichuan	Chongqing	Guizhou	Yunnan	Hubei	Total
Sichuan	****	0.011	0.038	0.036	0.001	0.022
Chongqing	22.477	****	0.030	0.043	0	
Guizhou	6.329	8.083	****	0.031	0	
Yunnan	6.694	5.564	7.815	****	0.013	
Hubei	249.750	/	/	18.981	****	
Total	11.114					

Note: PhiPT measure of genetic differentiation among populations; values closer to 0 indicate low differentiation. higher values indicate stronger connectivity. /: gene flow could not be estimated due to insufficient sample size or monomorphic loci. ****: self-comparison, not applicable.

**Table 7:** Nei’s genetic distance and genetic identity among regional *P. infestans* populations.

Populations	Sichuan	Chongqing	Guizhou	Yunnan	Hubei
Sichuan	****	0.982	0.956	0.971	0.965
Chongqing	0.019	****	0.961	0.957	0.969
Guizhou	0.045	0.040	****	0.949	0.952
Yunnan	0.030	0.044	0.052	****	0.954
Hubei	0.035	0.032	0.049	0.047	****

Note: Nei’s genetic identity: measure of allele similarity between populations; values closer to 1 indicate higher similarity. Nei’s genetic distance: measure of divergence between populations; higher values indicate greater differentiation. ****: self-comparison, not applicable.

## Data Availability

The original contributions presented in this study are included in the article/Supplementary Materials. Further inquiries can be directed to the corresponding author(s).

## Supplementary Materials

The following supporting information can be downloaded at https://www.mdpi.com/article/10.3390/jof12080553/s1. Figure S1. Regional distribution of Phytophthora infestans isolates included in the SSR analysis; Figure S2. Shared and unique SSR genotypes among mating-type groups of *P. infestans*; Figure S3. Regional distribution of mitochondrial-DNA haplotypes in *P. infestans* populations; Figure S4. Temporal distribution of virulence genes in *P. infestans* isolates; Figure S5. UPGMA of *P. infestans* isolates based on SSR genetic distance; Table S1. SSR primers used for genotyping *P. infestans* isolates.

## Abbreviations

The following abbreviations are used in this manuscript:

UPGMA	Unweighted Pair Group Method with Arithmetic Mean
SSR	Simple Sequence Repeat
SC	Sichuan
CQ	Chongqing
GZ	Guizhou
YN	Yunnan
HB	Hubei
