# Supplementary material for: High Genotypic Polymorphism and Extensive Gene Flow Drive the Population Dynamics of Potato Oomycete Pathogen, Phytophthora infestans, Across Southwestern China
Source: J Fungi (Basel). 2026 Jul 24;12(8):553. doi: 10.3390/jof12080553 (PMC13514179; doi:10.3390/jof12080553)
Supplement: Supplementary file 1 [file jof-12-00553-s001.zip › jof-4407119-supplementary.pdf]

Supporting Information's

High Genotypic Polymorphism and Extensive Gene Flow Drive the Population Dynamics of Potato Oomycete Pathogen, *Phytophthora* infestans Across Southwestern China

The following Supporting Information are available for this article:

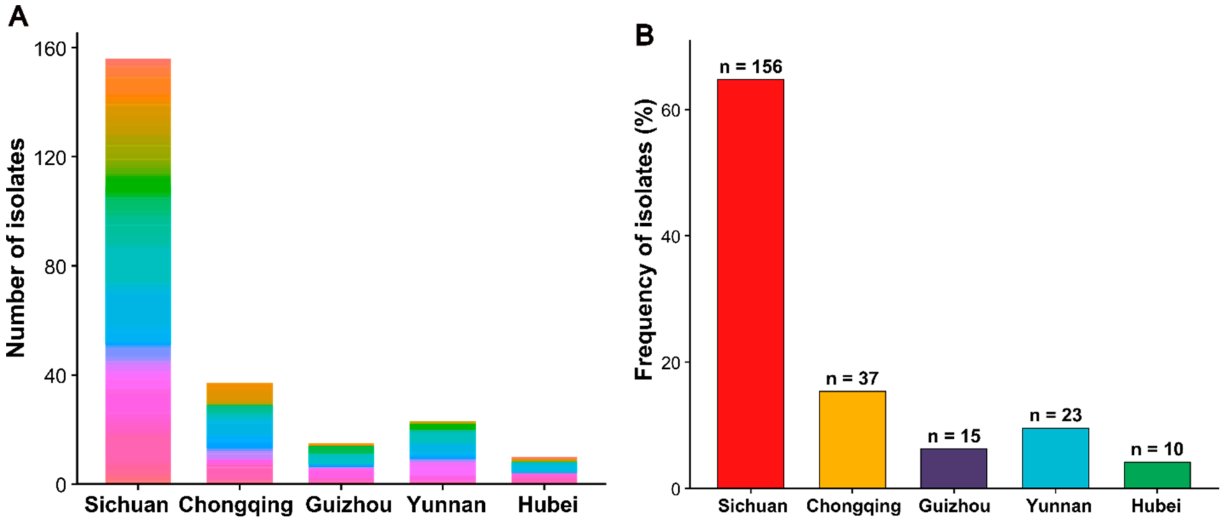

Figure S1. Regional distribution of *Phytophthora infestans* isolates included in the SSR analysis. (**A**) Regional composition of isolates according to multilocus SSR genotype. (**B**) Number and relative frequency of isolates collected from Sichuan, Chongqing, Guizhou, Yunnan and Hubei.

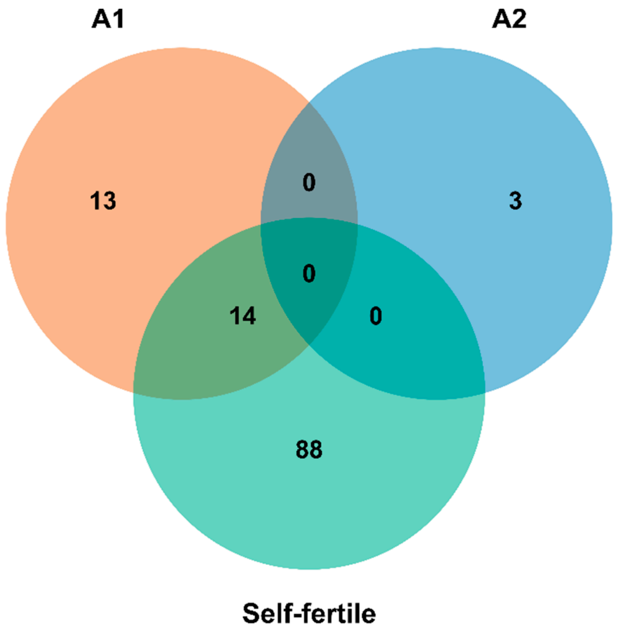

Figure S2. Shared and unique SSR genotypes among mating-type groups of *P. infestans*. The Venn shows the number of SSR genotypes unique to A1, A2 and self-fertile isolates, as well as genotypes shared among mating-type groups.

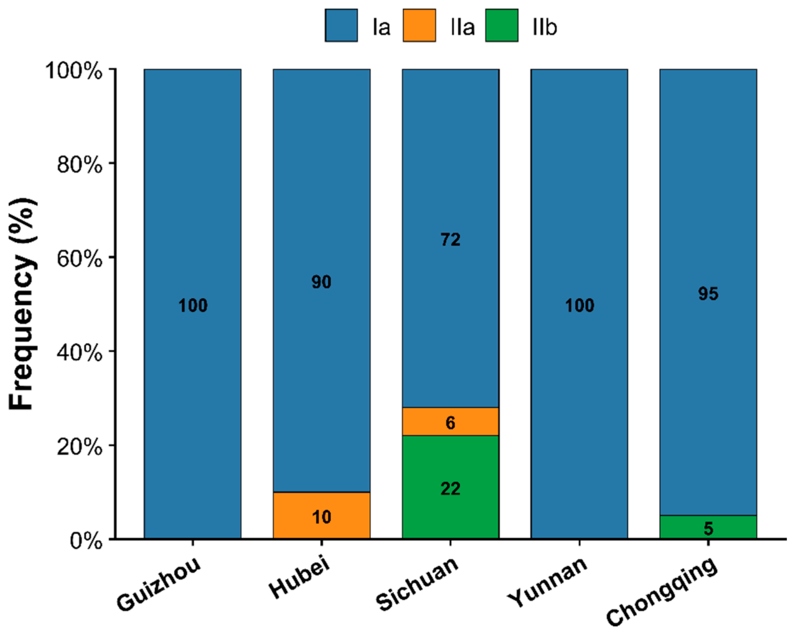

Figure S3. Regional distribution of mitochondrial-DNA haplotypes in *P. infestans* populations. Stacked bars show the relative frequency of mtDNA haplotypes Ia, IIa and IIb in each regional population.

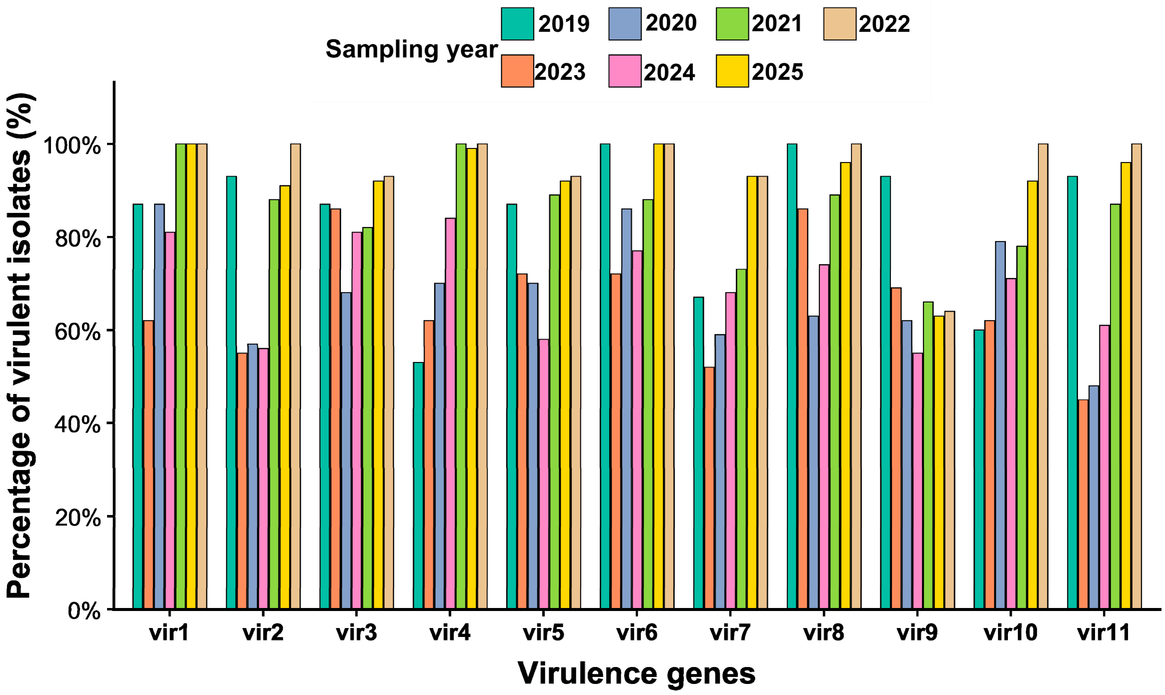

Figure S4. Temporal distribution of virulence genes in *P. infestans* isolates. The frequency of isolates carrying virulence genes vir1–vir11 is shown across sampling years. Bars represent the percentage of virulent isolates for each virulence gene in each year.

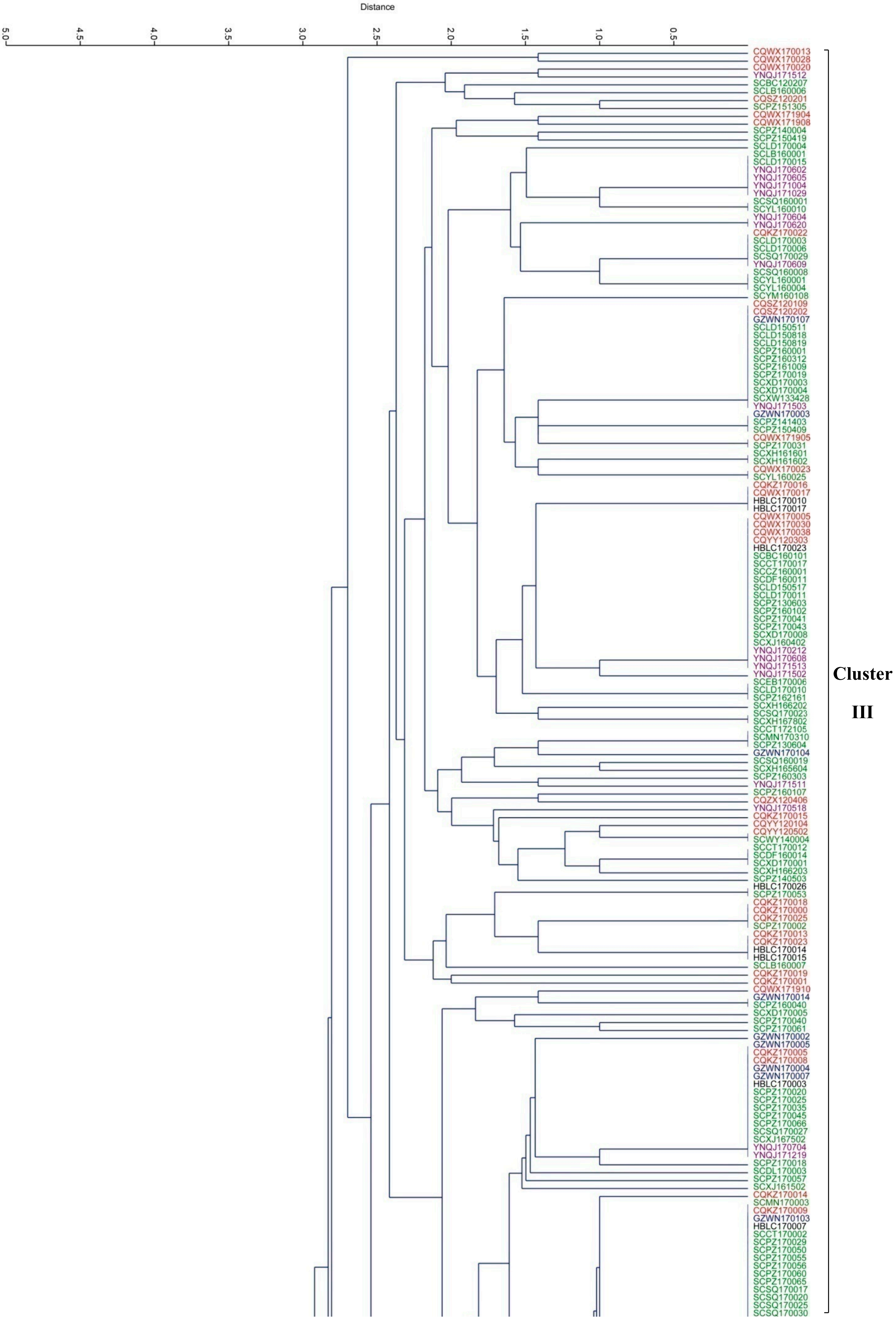
Distance
5.0
4.5
4.0
3.5
3.0
2.5
2.0
1.5
1.0
0.5
CQWX170013
CQWX170028
CQWX170020
YNQJ171512
SCBC120207
SCLB160006
CQSZ120201
SCPZ151305
CQWX171904
CQWX171908
SCPZ140004
SCPZ150419
SCLD170004
SCLB160001
SCLD170015
YNQJ170602
YNQJ170605
YNQJ171004
YNQJ171029
SCSQ160001
SCYL160010
YNQJ170604
YNQJ170620
CQKZ170022
SCLD170003
SCLD170006
SCSQ170029
YNQJ170609
SCSQ160008
SCYL160001
SCYL160004
SCYM160108
CQSZ120109
CQSZ120202
GZWN170107
SCLD150511
SCLD150818
SCLD150819
SCPZ160001
SCPZ160312
SCPZ161009
SCPZ170019
SCXD170003
SCXD170004
SCXW133428
YNQJ171503
GZWN170003
SCPZ141403
SCPZ150409
CQWX171905
SCPZ170031
SCXH161601
SCXH161602
CQWX170023
SCYL160025
CQKZ170016
CQWX170017
HBLC170010
HBLC170017
CQWX170005
CQWX170030
CQWX170038
CQYY120303
HBLC170023
SCBC160101
SCCT170017
SCCZ160001
SCDF160011
SCLD150517
SCLD170011
SCPZ130603
SCPZ160102
SCPZ170041
SCPZ170043
SCXD170008
SCXJ160402
YNQJ170212
YNQJ170608
YNQJ171513
YNQJ171502
SCEB170006
SCLD170010
SCPZ162161
SCXH166202
SCSQ170023
SCXH167802
SCCT172105
SCMN170310
SCPZ130604
GZWN170104
SCSQ160019
SCXH165604
SCPZ160303
YNQJ171511
SCPZ160107
CQZX120406
YNQJ170518
CQKZ170015
CQYY120104
CQYY120502
SCWY140004
SCCT170012
SCDF160014
SCXD170001
SCXH166203
SCPZ140503
HBLC170026
SCPZ170053
CQKZ170018
CQKZ170000
CQKZ170025
SCPZ170002
CQKZ170013
CQKZ170023
HBLC170014
HBLC170015
SCLB160007
CQKZ170019
CQKZ170001
CQWX171910
GZWN170014
SCPZ160040
SCXD170005
SCPZ170040
SCPZ170061
GZWN170002
GZWN170005
CQKZ170005
CQKZ170008
GZWN170004
GZWN170007
HBLC170003
SCPZ170020
SCPZ170025
SCPZ170035
SCPZ170045
SCPZ170066
SCSQ170027
SCXJ167502
YNQJ170704
YNQJ171219
SCPZ170018
SCDL170003
SCPZ170057
SCXJ161502
CQKZ170014
SCMN170003
CQKZ170009
GZWN170103
HBLC170007
SCCT170002
SCPZ170029
SCPZ170050
SCPZ170055
SCPZ170056
SCPZ170060
SCPZ170065
SCSQ170017
SCSQ170020
SCSQ170025
SCSQ170030
Cluster
III

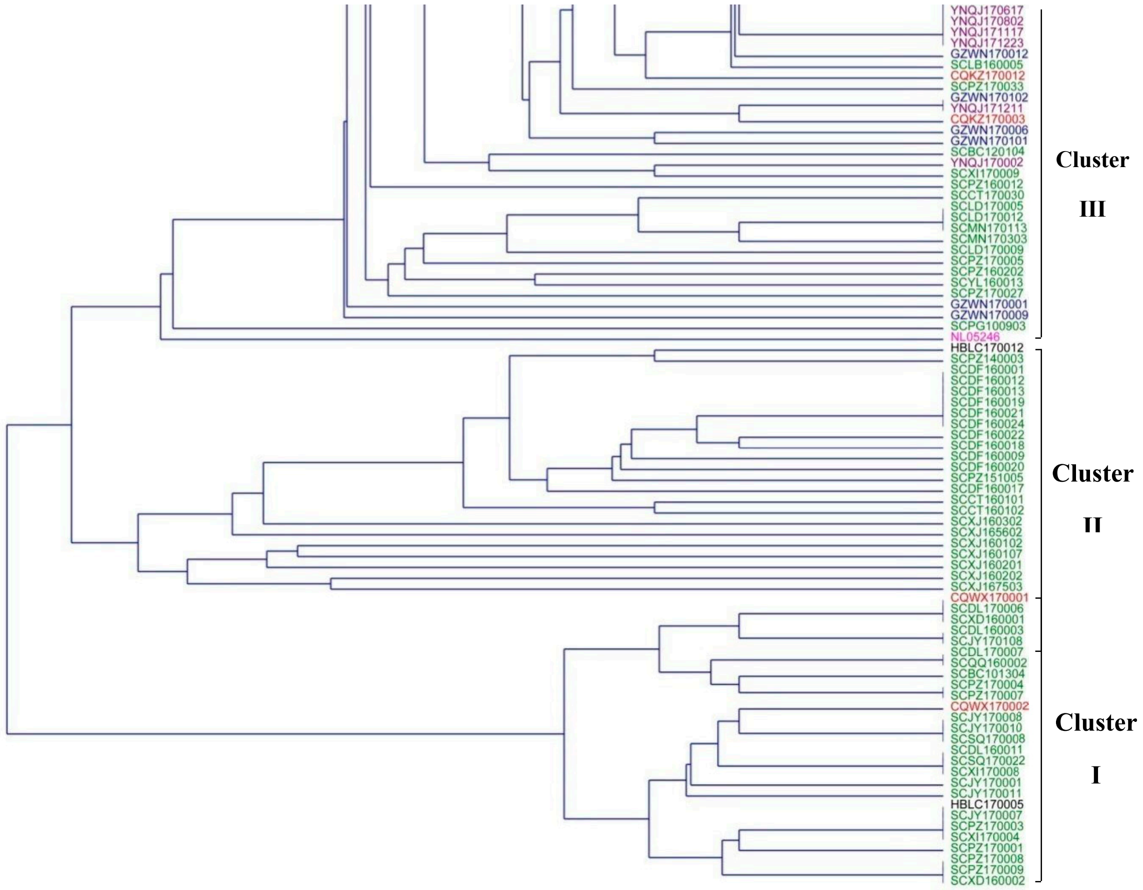

Figure S5. UPGMA of *P. infestans* isolates based on SSR genetic distance. The dendrogram was constructed using Euclidean distance and the unweighted pair-group method with arithmetic mean. Three major genetic clusters were identified and used for cluster-based genotype and phenotype comparisons.

Table S1. SSR primers used for genotyping *P. infestans* isolates.

Locus	Primer sequence	Product (bp)	Reference
**D13**	F:TGCCCCCTGCTCACTC<br>R:GCTCGAATTCATTTTACAGACTTG	100-185	[1]
**G11**	F:TGCTATTTATCAAGCGTGGG<br>R:TACAATCTGCAGCCGTAAGA	130-180	[1]
**Pi04**	F:AGCGGCTTACCGATGG<br>R:GTTTCAGCGGCTGTTTCGAC	160-175	[1]
**Pi26**	F:GCAGTAGCCGTAGTCCTCAG<br>R:GTTCCAAATCGTCAACCAAC	173-188	[2]
**Pi4B**	F:AAAATAAAGCCTTTGGTTCA<br>R:GCAAGCGAGGTTTGTAGATT	200-295	[1]
**Pi4G**	F:CGCTGTGTGGATGACAAGTA<br>R:TCGACCTGACATACGAGCTA	157-177	[3]
**Pi63**	F:ATGACGAAGATGAAAGTGAGG<br>R:CGTATTTTCCTGTTTATCTAACACC	265-280	[1]
**Pi70**	F:ATGAAAATACGTCAATGCTCG<br>R:CGTTGGATATTTCTATTTCTTCG	185-205	[1]
**SSR2**	F:CGACTTCTACATCAACCGGC<br>R:GTTTGCTTGGACTGCGTCTTTAGC	165-180	[1]
**SSR3**	F:ACTTGCAGAACTACCGCCC<br>R:GTTTGACCACTTTCCTCGGTTC	255-275	[1]
**SSR4**	F:TCTTGTTCGAGTATGCGACG<br>R:GTTTCACTTCGGGAGAAAGGCTTC	280-305	[1]
**SSR6**	F:GTTTTGGTGGGGCTGAAGTTTT<br>R:TCGCCACAAGATTTATTCCG	230-250	[1]
**SSR8**	F:AATCTGATCGCAACTGAGGG<br>R:GTTTACAAGATACACACGTCGCTCC	250-275	[1]
**SSR11**	F:TTAAGCCACGACATGAGCTG<br>R:GTTTAGACAATTGTTTTGTGGTCGC	325-360	[1]

Primer sequences, expected product sizes and reference sources are provided for the 14 SSR loci used in this study.

References

1. Li, Y.; Cooke, D.E.; Jacobsen, E.; van der Lee, T. Efficient multiplex simple sequence repeat genotyping of the oomycete plant pathogen Phytophthora infestans. *J Microbiol Methods* **2013**, *92*, 316-322, doi:10.1016/j.mimet.2012.11.021.
2. Lees, A.K.; Wattier, R.; Shaw, D.S.; Sullivan, L.; Williams, N.A.; Cooke, D.E.L. Novel microsatellite markers for the analysis of Phytophthora infestans populations. *Plant Pathology* **2006**, *55*, 311-319, doi:https://doi.org/10.1111/j.1365-3059.2006.01359.x.
3. Knapova, G.; Schlenzig, A.; Gisi, U. Crosses between isolates of Phytophthora infestans from potato and tomato and characterization of F1 and F2 progeny for phenotypic and molecular markers. *Plant Pathology* **2002**, *51*, 698-709, doi:https://doi.org/10.1046/j.1365-3059.2002.00762.x.
